# Understanding care relationships in diabetes practice: A psychodynamic interview-based exploratory study

**DOI:** 10.1371/journal.pone.0263226

**Published:** 2022-02-17

**Authors:** Francesco Marchini, Andrea Caputo, Viviana Langher, Chiara Giuliani, Alessio Convertino, Rossella Mazzilli, Antongiulio Faggiano, Angela Napoli

**Affiliations:** 1 Department of Dynamic and Clinical Psychology, “Sapienza” University of Rome, Rome, Italy; 2 Italian Center of Analytical Psychology, CIPA, Roma, Italy; 3 Endocrinology Unit, Department of Clinical and Molecular Medicine, “Sapienza” University” of Rome, Rome, Italy; GITAM College of Pharmacy: GITAM Institute of Pharmacy, INDIA

## Abstract

**Objective:**

To explore the subjective experience of physicians working in diabetic settings about their care relationships in order to find some unique clues contributing to physician professional health and capacity to manage patients’ adherence.

**Research design and methods:**

An interview-based exploratory study has been carried out involving 18 physicians (77.8% female) with at least 3 years of clinical practice in diabetes care. In-depth interviews about the emotional experience with patients with diabetes were conducted and audio recorded. Interviews transcripts were analyzed through a computer-based text analysis which allowed the identification of thematic domains (Cluster Analysis) and latent factors (Correspondence Analysis) viewed through a psychodynamic and constructivist lens.

**Results:**

Six thematic domains emerged respectively referring to: Concern (8.43%), Control (14.42%), Ambivalence (22.08%), Devotion (22.49%), Guilt (19.29%) and Strive for Achievement (13.30%). Moreover, three latent dimensions were taken into account, which explained 69.20% of data variance: Affect Repression (28.50%), Tendency to Repair (22.70%) and Anxiety Pattern (18.00%).

**Conclusions:**

Overall, the results of the present study confirm the challenging nature of diabetes care. In particular, physicians ongoing effort to restore patients’ psychological integrity in chronic condition constitute the most novel finding above all. In this regard, the need for emotional labor in physicians’ education and training is suggested in order to both prevent burnout symptoms (e.g. depersonalization) and promote shared decision making in care relationships. However, findings should be treated as preliminary given the convenience nature of the sample and its reduced size.

## Introduction

Diabetes is a challenging disease not only for patients and their relatives but also for those responsible for their care. To provide the most effective care and successfully address the needs of patients, physicians are often exposed to fatigue as well as both mental and physical problems [1]. Most of all, patient nonadherence constitutes a pervasive threat to health and clinical outcomes, thus making diabetes care a “relational challenge” in motivating patients to self-care.

In this regard, a recent study showed 47% of endocrinologists were emotionally exhausted and reported burnout symptoms [2]. Although only a few studies investigated health status in physicians working in diabetic settings, it has been showed that diabetes and pre-diabetes care are associated with worse mental health and higher levels of burnout [3, 4]. Burnout syndrome consequences include stress, depression and suicide risk [5] as well as physician dropout rate and reduced empathy with patients, leading to clinical errors, worse medical outcomes and care dissatisfaction [2, 6, 7].

Qualitative research focusing on diabetologists’ subjective experience highlighted feelings of frustration connected to patient nonadherence, worries about failure to achieve clinical outcomes and an overinvolvement with patient accompanied by the fear to be overwhelmed [8–11]. Moreover, physicians working in diabetic settings reported the sensation of not having enough psychological resources to address patients’ needs [12] and tolerate emotional labor in healthcare relationships [9, 13]. Environmental factors also emerged (e.g. time constraints, lack of specialized personnel) as barriers to personalized care [9, 10, 12].

These pieces of research demonstrate that care relationships in diabetes can represent an emotional burden for physicians. Recently, Craven et al. [8] proposed the term “diabetes distress”–usually referring to patient distress connected with diabetes–to indicate a specific feature of diabetes professional experience that needs to be more deeply explored and consequently addressed by specific interventions to improve quality of care. Although–as yet highlighted–there are some studies addressing the subjective experience of physicians working in diabetic settings, none of them has examined the relationship with patients with diabetes from a psychodynamic point of view [14]. In this perspective, interpersonal experiences (e.g. relation with patients with diabetes) are internalized as symbolic representations involving feelings, images, sensations, expectations and beliefs about the relationship with the other that drive–often in an implicit way–interpersonal interactions. In line with this, emotions experienced within relationships are derived from “affective symbolizations” [15], which can be shared from people who live or work in the same context (e.g. physician working in diabetic settings). Affective symbolizations can emerge through narratives and discourses, looking at language and words as social vehicles for communicating the internal world. On this premise, it is important to investigate how physicians unconsciously symbolize patients with diabetes to more deeply understand critical issues and potential relational skills that can be further promoted in such a medical setting.

The purpose of the present study was to explore the subjective experience of physicians working in diabetic settings about their care relationships as to grasp their affective symbolizations. This may allow the detection of relational issues that can be crucial for promoting professional health of physicians working in diabetic settings, with fruitful advantages for patient adherence to treatment. In this way, the study aims at finding some unique clues contributing to physician capacities to manage patient adherence and elaborate on the emotional labor of clinical care of persons with diabetes.

## Research design and methods

### Participants and procedures

The study participants were physicians working in two public Italian hospitals located in urban areas, dealing with both outpatient and inpatient diabetes care. The inclusion criteria were at least three years of clinical experience with patients with diabetes and availability to be interviewed. Overall, the study sample included 18 participants, mostly females (77.8%) and with a mean age of 44.11 years (SD = 15.16), with an adequate size for interview-based studies in terms of information power [16]. All physicians treated patients with both type 1 and type 2 diabetes. Specifically, participants were administered an audio-recorded structured interview in order to examine their deep feelings about caring for patients with diabetes. The interview consisted of open-ended questions overall aimed at facilitating associative processes about their work experience. The questions focused on three different areas: subjective perceptions about their care relationship with patients with diabetes [1], the most significant episodes respectively dealing with successful and unsuccessful professional experiences with such patients [2], and suggestions for improving care relationships within diabetic settings [3]. All the interviews (each lasting 40 minutes on average) were conducted by an external psychology researcher at physicians’ workplace, in a reserved and confidential environment. The study was conducted according to the Declaration of Helsinki (1964) and the principles of the institutional review board (ethical approval was not required as the research was not a medical nature and there were no potential risks for participants). The participation in research was voluntary and a written informed consent was provided for all the physicians. All the data were analyzed anonymously.

### Textual data analysis

Emotional Text Analysis [17] was used as research framework to get a representation of narrative contents through few and significant thematic domains, in line with previous research on healthcare professionals [18, 19]. In line with the double reference principle of language [20], assuming both a lexical-cognitive and a symbolic-affective function of words, emotional meanings can be derived from semantic isotopies of narratives rather than from categories previously established by the researcher. Indeed, word co-occurrence is hypothesized to detect the syntagmatic relations between parts of discourse, through deconstructing the typical structuring or ordered constituent parts of language, thus grasping more implicit and symbolic processes [17], consistent with the psychoanalytic principle of free association. To this purpose some statistical multidimensional techniques are carried out through text analysis software, in our case T-Lab [21]. Specifically, Cluster Analysis (CA) allows grouping sentences based on their co-occurring words from a digital “presence-absence” matrix (with sentences in rows and words in columns, respectively), thus detecting thematic domains. Besides, Multiple Correspondence Analysis (MCA) enables the exploration of the relationship between such domains in a multi-dimensional space, thus detecting some latent factors [21]. Each domain is analyzed based on its typical vocabulary (words co-occurring with highest probability, evaluated through Chi-square test) and clustered extracts giving direct voice to participants. Whereas, each factor is analyzed from the clusters that are most associated with its positive and negative pole (based on the absolute contributions of clusters to the formation of each factor) [22, 23]. Therefore, both clusters and factors are labeled by the researcher, with the former representing different meaningful themes emerging from narratives and the latter being conceived as underlying dimensions explaining for the potential coexistence of such themes. The interpretation process is based on a constructivist and psychodynamic paradigm [24, 25] and on the use of models of affective symbolization [17] referred to affective-motivational dynamics such as affiliation (e.g., feelings of inclusion/exclusion), power (e.g., feelings of control/dependence), and achievement (e.g., feelings of success/failure), which may reveal basic sense-making processes.

## Results

Cluster analysis has detected six thematic domains. In Table 1, the percentage of sentences grouped in each cluster as well as the most characteristic keywords and some examples of participants’ extracts are reported.

**Table 1:** Clusters.

Cluster 1		Cluster 2		Cluster 3		Cluster 4		Cluster 5		Cluster 6	
*Concern* (8.43%)		*Control* (14.42%)		*Ambivalence* (22.08%)		*Devotion* (22.49%)		*Guilt* (19.29%)		*Strive for achievement* (13.30%)	
*Word*	*χ²*	*Word*	*χ²*	*Word*	*χ²*	*Word*	*χ²*	*Word*	*χ²*	*Word*	*χ²*
Relationship	199.85	Insulin	247.32	To become attached	193.02	Outpatient	154.32	Diabetes	296.22	To try	181.00
Patient	92.76	Therapy	107.42	Role	152.37	Diabetology	152.65	Difficult	293.14	To achieve	123.94
Physician	52.85	Glucose	74.11	Bad	87.93	Work	70.03	To feel	208.30	Gratification	61.03
Time	39.33	Eating	70.08	To get angry	55.41	Specialization	69.74	Guilt	13.72	Problem	35.85
To phone	34.30	Lifestyle	30.76	Wrong	46.60	Endocrinology	66.21	Closeness	11.00	To help	33.75
To care for	28.48	To manage	23.92	Boundary	30.53	Profession	65.45	Discomfort	10.70	To solve	32.45
Trust	18.29	Disease	23.24	People	17.53	Year	50.26	Frustration	7.44	To fix	19.11
To dedicate	24.44	To monitor	15.81	To allow	13.21	Medicine	39.15	Sufferance	7.01	Effort	18.91
To welcome	14.09	Complication	14.45	To pay	7.01	Experience	31.19	Diagnosis	4.54	To hope	16.05
Human	8.17	Risk	7.23	Affection	5.52	Training	18.94	To die	4.02	Complicity	9.24
Examples of elementary context units											
*I would have more time to dedicate to patients*, *without other people knocking on the door*		*If patients suddenly stop taking their insulin*, *the risk is very great for disease and complications*		*I am a person who becomes attached to patients*, *even if I tend to defend myself in many things*		*I have been working in a diabetes outpatient clinic for many years now and I can say that I have had the experience*		*I once felt guilty because I made a patient cry by asking her so many questions about how she was managing her diabetes*		*I felt contentment and gratification because we were able to make the therapy work with a patient who also had a pituitary problem*	
*To care for patients it is important to welcome and build a trusting relationship with them*		*There are many things to monitor*, *such as therapy*, *glucose*, *eating*, *lifestyle*, *which need to be effectively managed*		*It is important that the roles are separated even if you enter people’s lives*, *I am a doctor and I am not just a friend*		*I have been in love with endocrinology since I was a student*, *I think it is one of the most fascinating branches of medicine*		*Sometimes I feel a strong closeness with patients to the point that their frustration becomes mine*		*A relationship of complicity is created with the patient in which one takes charge of overcoming obstacles and solving problems*	
*You put yourself into the physician-patient relationship as a human being with all your facets*		*I had an obese patient with very high levels of glucose despite the therapy*, *then I found out that he was lying about his eating*		*Often patients get angry with you even if you has nothing to do with it and they are the ones who are wrong*		*The outpatient approach in diabetology was a formative training for me because it gave me a broader view on the patient*		*It was difficult to communicate a diagnosis of diabetes to a very young pregnant girl with whom I felt strong emotional closeness*		*It is important to try to understand why a person behaves in a certain way and help him or her better so to fix things*.	

Note: The threshold value of Chi-square test (χ²) for each lemma is 3.84 (df = 1; p = 0.05). Textual data were translated into English only for the purposes of the paper.

### Thematic domains

#### Cluster 1: Concern

This cluster expresses the feeling of concern in caring for patients, which is mostly associated with the lack of time and appropriate setting to deliver personalized care. The physician-patient relationship seems to represent the most relevant aspect of effective diabetes care. In particular, patients’ needs of being listened to and establishing a human and trustful bond are highlighted.

#### Cluster 2: Control

This cluster emotionally evokes an underlying feeling of distrust in care relationships and the urge to control them. On the one hand, physicians express hypervigilance in front of diabetes complications, which require constant diabetes monitoring. On the other hand, the risk of patient nonadherence to medical treatment and reduced care engagement is reported.

#### Cluster 3: Ambivalence

This cluster expresses ambivalent feelings regarding care relationships. Specifically, physicians perceive a strong affective attachment to patients that may lead to overinvolvement and fear to lose their professional role. As a consequence, there is the need to define relational boundaries to defend themselves and avoid getting in touch with patients’ frustration about diabetic care.

#### Cluster 4: Devotion

This cluster deals with a strong devotion in terms of love and loyalty for the specific field of diabetes care as well as enthusiasm and proudness with regard to work practice. The exclusive focus on work significance and meaningfulness rather than on care relationships seems to suggest an anchoring to professional mission, involving an ideal and intellectualized vision of patients.

#### Cluster 5: Guilt

This cluster expresses feelings of guilt and inadequateness in facing several difficult situations about communicating a diagnosis of diabetes and managing care more widely. Physicians are overwhelmed by patients’ discomfort and emotional sufferance, which they feel partially responsible for, since diabetes is a long-lasting chronic condition that cannot be definitively cured.

#### Cluster 6: Strive for achievement

This cluster highlights the tendency to spend effort in caring for patients with diabetes, which is regarded as a challenging task. Physicians focus on successful and collaborative experiences with patients that generate feelings of gratification and contentment. Problem-solving and achievement orientation are deemed as ensuring a sense of efficacy and hope to alleviate patients’ burden.

### Latent factors

The first three latent dimensions emerging from MCA have been taken into account, which overall explain 69.20% of data variance. The absolute contributions of clusters to the factorial axes are presented in Table 2.

**Table 2:** Absolute contributions of clusters to each factor.

	Factor 1	Factor 2	Factor 3
	*Work meaningfulness*	*Tendency to repair*	*Anxiety pattern*
Cluster 1			
*Concern*	.02 (-)	.12 (-)	.53 (-)
Cluster 2			
*Control*	.14 (+)	.00 (+)	.02 (-)
Cluster 3			
*Ambivalence*	.03 (+)	.10 (-)	.26 (+)
Cluster 4			
*Devotion*	.62 (-)	.02 (+)	.09 (+)
Cluster 5			
*Guilt*	.10 (+)	.56 (+)	.00 (+)
Cluster 6			
*Strive for achievement*	.09 (+)	.20 (-)	.10 (+)

Note: The sign reported in brackets (-/+) indicates the specific factorial pole (negative/positive) associated with each cluster.

### Affect repression (F1)

The first factor (28.50% of total variance) opposes cluster 4 to cluster 2 and seems to refer to a defensive tendency aimed at lessening potential conflicts in care relationships through focusing on self-assertiveness and rational thinking. This is expressed through an intellectualized view of diabetology practice (Cluster 4) and logic explanations to justify potential unresponsiveness to treatment (Cluster 2). Therefore, this factor synthesizes an affect repression through resorting to technical power and expertise to minimize the risk of self-perceived uselessness and distress.

### Tendency to repair (F2)

The second factor (22.70% of total variance) opposes cluster 6 to cluster 5 and seems to deal with the tendency to repair, intended as the effort to restore patients’ integrity despite the disruptiveness of diabetes. Strive for achievement and sense of hope about a successful care management emerge (Cluster 6), which are accompanied by a profound sense of responsibility and empathic identification with patients’ emotional sufferance (Cluster 5). Therefore, this factor synthesizes the compassion and desire to care for patients as to establish a satisfactory relationship with them.

### Anxiety pattern (F3)

The third factor (18.00% of total variance) opposes cluster 1 to cluster 3 and seems to suggest an anxiety pattern characterized by ambivalent feelings about the desire for closeness and contact with patients and the need to distance oneself from them. Worries about ensuring trustful and effective care relationships (Cluster 1) are intertwined with the need for maintaining professional role and avoiding excessive closeness (Cluster 3). Therefore, this factor synthesizes the concerns about providing personalized and humanized care and the consequent fear to be overinvolved in care relationships.

## Conclusions

Six thematic domains have been detected, which shape affective symbolizations about caring for patients with diabetes and respectively referred to concern (8.43%), control (14.42%), avoidance (22.08%), devotion (22.49%), guilt (19.29%), and strive for achievement (13.30%). Such domains are conceived along three latent dimensions synthesizing the physicians’ subjective experience.

The first factor deals with a dynamic of affect repression suggesting a defensive minimization of potential perceived distress in care relationships by resorting to intellectualization and rationalization. Several studies have demonstrated that physicians tend to develop emotional detachment when dealing with stressful situations as to maintain scientific and medical objectivity [26–28]. Indeed, diabetes care can progressively lead to burnout because of several care challenges involving frustration about patients’ nonadherence to recommendations and emotional fatigue about the redundancy of treatment topics [3, 4, 8]. In order to protect oneself, such overwhelming emotions are handled by focusing on more technical and biomedical aspects concerning the disease [29], seen as something separated from patients in line with the “phallocentric function” of medical intervention proposed by Fornari [20]. The use of mature defenses allows alleviating discomfort and anxiety, as found in other studies about healthcare professionals [18, 30], as to reassure oneself and avoid feelings of powerlessness. In our results, this is expressed through abstract self-serving explanations about the value of professional mission or hypervigilant and controlling behaviors in diabetes management. However, some authors suggest that such strategies may reinforce depersonalization and reduce job satisfaction over time [27, 31].

The second factor deals with the tendency to repair patients’ integrity as “persons” since diabetes is a chronic and disabling condition involving ongoing psychological adjustment due to the impossibility to restore a completely healthy state [32]. Healthcare providers are thus faced with a potential empathic identification with care recipients, which may lead to a continuous effort to repair patients’ damaged self in symbolic terms [33–35]. Moreover, since diabetes care consists in a challenging step-by-step path involving monitoring and adherence to medical treatment, patients may enact denial strategies to handle disease related burden that prevent them from adopting self-care behaviors [10, 32]. This in turn affects care relationships, potentially engendering feelings of guilt and inadequateness in physicians and consequently the urge to address patients’ needs [36]. Indeed, previous studies have found a strong perceived responsibility in dealing with the emotional sufferance of patients with diabetes [8, 12] as well as a sense of deficiency and personal failure [9, 35] that can be overcome by using active listening and relational skills [13, 29, 37]. In this sense, both the fear of failure and the strive for achievement are strongly intertwined and underlie a motivation to derive satisfaction from the mastery of challenging tasks and effective care relationships [38].

The third factor deals with an anxiety pattern shaping the physician-patient interaction, characterized by insecure-ambivalent attachments. As reported by several pieces of research, physicians treating patients with diabetes experience feelings of anxiety [8, 9, 12, 37], with specific regard to worries about patients’ outcomes, fears of losing control over treatment and time constraints limiting individualized care. This may lead to feeling overwhelmed by social needs of patients as well as confused about one’s professional role because of the risk of an excessively symbiotic relation with patients [8]. As a consequence, physicians strive to maintain professional boundaries and try to get less involved in patients’ emotional concerns [29], for instance adopting an assertive attitude and paternalistic communication with patients within an asymmetrical relationship [8].

The limitations of the present study include the convenience nature of the sample and its reduced size that prevent from any generalization of the findings. Besides, the lack of secondary subgroup analyses by participants’ characteristics (e.g., gender, professional tenure, psychological status) does not allow considering some potential variability in the subjective experience of physicians working in diabetic settings. From this perspective, this study should be considered as explorative and preliminary, providing some qualitative clues that contribute to the understanding of care relationships. Enrolling a larger sample of physicians working in hospitals and/or GPs outpatient clinics throughout the country could make the present findings more robust and of impact in scientific literature. Overall, our results confirm the challenging nature of diabetes care [39]. Indeed, the ongoing effort to restore patients’ psychological integrity in such a chronic condition may lead to distress and professional fatigue. In this regard, physicians’ potential feelings of guilt concerning the impossibility to completely achieve healing—as a novel finding compared to previous studies—could be further explored in future research. In sum, the study highlights the need for emotional labour regarding physician-patient interaction to avoid maladaptive strategies in managing care relationships. On the one hand, the overuse of defensive mechanisms aimed at affect repression may involve the risk of depersonalization and detachment in relating to patients. This requires the development of affective and relational skills in physicians’ education and training. On the other hand, perceived uncertainty in care relationships could imply the enactment of paternalistic or conflictual behaviors, assuming a monopoly on decision-making about care. Therefore, reflective practices and clinical supervision should be promoted to adopt an integrative function in physician–patient relationship, intended as the capacity to activate a shared-decision making. This could make patients more prone to participate in and adhere to treatment as well as activate their personal resources for self-care [40–42].

## Supporting information

S1 File Interview guide. (DOCX)
