# Supplementary material for: Understanding care relationships in diabetes practice: A psychodynamic interview-based exploratory study
Source: PLoS One. 2022 Feb 17;17(2):e0263226. doi: 10.1371/journal.pone.0263226 (PMC8853562; doi:10.1371/journal.pone.0263226)
Supplement: S1 File — (DOCX) [file pone.0263226.s001.docx]

**Interview Guide**

1. You can start by describing your experience working with people with diabetes. When did it start and which are the roles did you take? What does your work with people with diabetes consist of? Why did you decide to specialize in this area?

2. Please take your time. Now think about your overall work experience. I am asking you to choose 5 adjectives or nouns which can reflect the relationship you have had with your patients in your work experience from the beginning until today. I know you may need some time so take all the time you need.

*Per each word*: To describe your relationship with patients you used the expression "...". Could you tell me an episode, an event, specifying one associated situation which could help me understand why you chose the word "…".

3. Please, now choose an episode in which you have felt particularly in difficulty with a patient, retracing your experience, and, if possible tell me about it.

4. Well, now I ask you to tell an episode in which you felt, on the contrary, particularly effective in your intervention with patients.

5. Is there any patient you have felt more connected to and why?

6. In general, how do you think your experience with patients may have influenced your professional practice? Are there any specific episodes in the relationship with patients that have markedly influenced your professional practice?

7. How do you think you can improve the relationship with patients, where it is most difficult?
